# Supplementary material for: Association between oral microbiome and seven types of cancers in East Asian population: a two-sample Mendelian randomization analysis
Source: Front Mol Biosci. 2023 Nov 21;10:1327893. doi: 10.3389/fmolb.2023.1327893 (PMC10702768; doi:10.3389/fmolb.2023.1327893)
Supplement: Supplementary file 1 [file DataSheet1.ZIP › Supplementary Materials/MR plots for tongue/tongue═╝/Ovarian cancer/pheno.1073_to_ovarian cancer_forest.pdf]

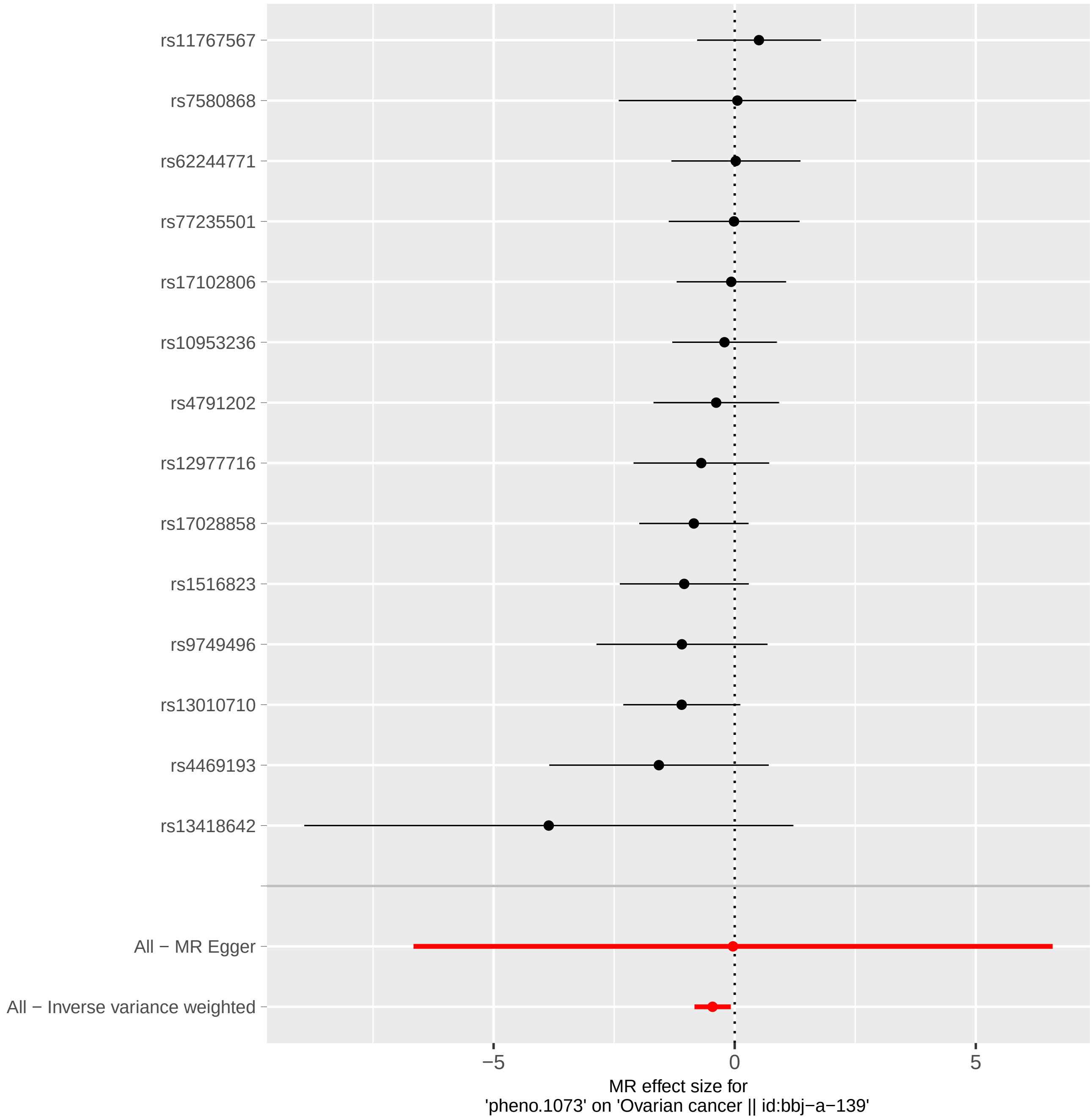
rs11767567
rs7580868
rs62244771
rs77235501
rs17102806
rs10953236
rs4791202
rs12977716
rs17028858
rs1516823
rs9749496
rs13010710
rs4469193
rs13418642
All − MR Egger
All − Inverse variance weighted
−5
0
5
MR effect size for
'pheno.1073' on 'Ovarian cancer || id:bbj−a−139'
